# Clerodendranoic Acid, a New Phenolic Acid from *Clerodendranthus spicatus*

**DOI:** 10.3390/molecules171113656

**Published:** 2012-11-19

**Authors:** Qingxia Zheng, Zhaocui Sun, Xiaopo Zhang, Jinquan Yuan, Haifeng Wu, Junshan Yang, Xudong Xu

**Affiliations:** 1 Institute of Medicinal Plant Development, Chinese Academy of Medical Sciences and Peking Union Medical College, Beijing 100193, China; Email: zhengqingxia916@126.com (Q.Z.); flydancingsun@163.com (Z.S.); xiaopozhang@yahoo.com (X.Z.); hfwu@implad.ac.cn (H.W.); jsyang@implad.ac.cn (J.Y.); 2 National Engineering Laboratory of Southwest Endangered Medicinal Resources Development, National Development and Reform Commission, Guangxi Botanical Garden of Medicinal Plant, Nanning 530023, China; Email: yjqgx@163.com

**Keywords:** *Clerodendranthus spicatus*, Lamiaceae, phenolic compounds

## Abstract

Phenolic acid derivatives are typical constituents of *Clerodendranthus spicatus* which were considered to the active principles of this medicinal plant. These chemical constituents with their interesting frameworks and biological significance attracted our attention. As part of our ongoing chemical investigation of *C. spicatus* using various column chromatography techniques, a new phenolic compound, named clerodendranoic acid (**1**), was isolated from the aerial parts of *C. spicatus* together with five known ones, including rosmarinic acid (**2**), methyl rosmarinate (**3**), caffeic acid (**4**), methyl caffeate (**5**), ethyl caffeate (**6**). Their structures, including stereochemical configurations, were completely established by extensive spectroscopic methods, mainly inclvolving 1D, 2D NMR, as well as HRESIMS.

## 1. Introduction

*Clerodendranthus spicatus* (Thunb.) C. Y. Wu (Lamiaceae) (*Orthosiphon stamineus* Thunb.), a popular folk medicine (also known as “Shen Cha” in Chinese), is distributed widely in the southern part of China and has been traditionally used for the treatment of nephritis, cystitis, rheumatism, *etc.* [1,2]. So far, many reports on chemical constituents of this species have been documented, demonstrating the presence of flavonoids [3], diterpenes [4,5,6,7,8], phenolic acids [3,9,10] and alkyl glycosides *etc*. [11], In particular, phenolic acid derivatives were identified as its typical constituents and were considered to the active principles of this medicinal plant. These rosmarinic acid derivatives, with their interesting frameworks and biological significance, have attracted our continuous attention. In present study, we obtained a new rosmarinic acid derivative, named clerodendranoic acid (**1**), along with five known compounds, rosmarinic acid (**2**), methyl rosmarinate (**3**), caffeic acid (**4**), methyl caffeate (**5**) and ethyl caffeate (**6**), from the aerial parts of *C*. *spicatus*. Herein, the isolation and structural elucidation of the new compound was described. This study not only deals with the spectral data of the new compound, but also enriches the phenolic acid derivatives obtained from *C. spicatus*, thereby providing a candidate for further study.

## 2. Results and Discussion

**Figure 1:**
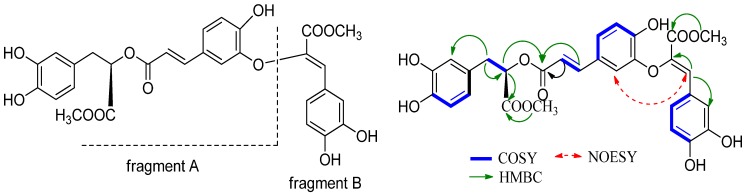
Structure and selected HMBC, COSY and NOESY correlation of **1**.

**Figure 2:**
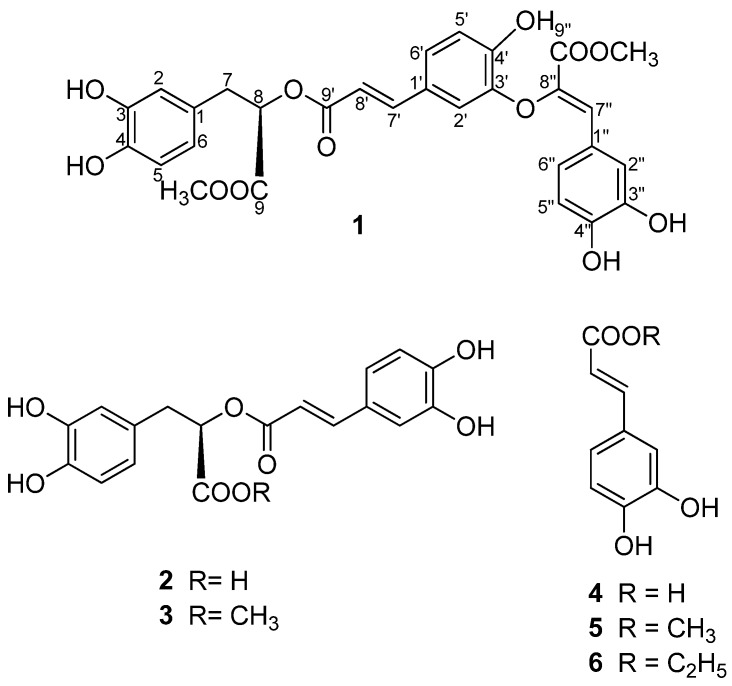
Structures of compounds **1**–**6**.

Compound **1** was isolated as a brown powder and its molecular formula (C_29_H_26_O_12_) was determined from the pseudo-molecular ion peak at *m/z* 589.1321 [M+Na]^+^ (calcd. 589.1322 [M+Na]^+^) obtained by HR-ESI-MS, consistent with seventeen degrees of unsaturation. Its IR spectrum exhibited bands for a hydroxyl group (3402 cm^−1^), an ester group (1695 cm^–1^) as well as characteristic absorptions of an aromatic ring (1602, 1,439 and 1020 cm^–1^). The ^1^H-NMR spectrum exhibited two methoxy group signals at *δ*_H_ 3.67 (s, 3H) and 3.74 (s, 3H). Two low-field olefinic proton signals were observed at *δ*_H_ 6.17 (d, *J* = 14.2Hz, H-8'), *δ*_H_ 7.50 (d, *J* = 14.2 Hz, H-7′) due to a *trans*-double bond conjugated with a carbonyl group [12]. Detailed analysis of the both the chemical shifts of H-7′ and H-8′ and their coupling constants suggested that they were the α and β protons of the *trans*-double bond. Additionally, three sets of ABX signals observed were attributed to phenyl rings according to their chemical shifts and coupling constants. Three aliphatic protons (*δ*_H_ 5.16, 3.03, 2.99) and the signals at *δ*_H_ 6.68 (br s), 6.67 (d, 7.8Hz), 6.54 (br d, 7.8) suggeseting the presence of a 3-(3,4-dihydroxyphenyl) lactic acid moiety, which were the characteristic signals of rosmarinic acid (**2**). Signals resonated at *δ*_H_ 7.31 (br s), 6.76 (d, 7.8Hz), 7.11 (br d, 7.8Hz) and *δ*_H_ 7.35 (s) indicated the presence of a caffeic acid unit with an oxygen atom attached to C-8′′ in **1** [13]. While the singlet signal at *δ*_H_ 7.35 showed that the olefinic geometrie of C-7′′-C-8′′double bond is *Z* [14]. The above structural elucidation was undoubtedly confirmed by ^13^C-NMR (APT) and HSQC spectral data (Table 1). Moreover, the positions of three carboxyls were determined according to the HMBC correlations from H-8 to carbons at C-9 at *δ* 165.9, H-7′ to carbon at *δ* 168.2 and H-7′′ to *δ* 172.4, respectively. Therefore, the structure of clerodendranoic acid contains a methyl caffeate moiety (fragment A) and a methyl rosmarinate (fragment B). The linkage between fragment A and B was determined on basis of observed cross-peak between H-2′ and H-7′′ in the NOESY experiment, suggesting the two fragments were connected with each other by an oxygen atom at C-3′ (Figure 1). Furthermore, the changes of ^13^C-NMR spectral data of C-3′ and C-4′ were also in line with the substituent parameter rules to the values reported by Dapkevicius *et al*. [13], typically the significant downfield shift of C-4′ (>150 ppm) and C-6′ (=125 ppm). Rosmarinic acid and rosmarinic acid derivatives as well as compound **1** obtained in the present research displayed similar optical rotations. Therefore, the absolute configuration at C-8 in **1** was supposed to be *R* from the viewpoint of biogenetic considerations. Thus, the structure of **1** was identified as 3′-*O*-(8′′-*Z*-methylcaffeoyl)-rosmarinic acid methyl ester as shown in Figure 2. Compound **1** was a new compound and was given the name clerodendranoic acid.

**Table 1:** ^1^H- and ^13^C-NMR data of compound **1** in CD_3_OD (*δ* in ppm, *J* in Hz).

Position	*δ*_C_	*δ*_H_	Position	*δ*_C_	*δ*_H_
1	128.9	-	6′	125.9	7.20 (br d, 7.8)
2	117.7	6.68 (br s)	7′	147.3	7.50 (d, 14.2)
3	146.3	-	8′	115.2	6.17 (d, 14.2)
4	145.6	-	9′	168.2	-
5	116.7	6.67 (d, 7.8)	1′′	126.1	-
6	122.0	6.54 (br d, 7.8)	2′′	118.2	7.31 (br s)
7	38.1	2.99 (dd, 8.4, 14.4)	3′′	146.7	-
		3.03 (dd, 4.8, 14.4)			
8	74.9	5.16 (dd, 4.8, 8.4)	4′′	149.2	-
9	173.4	-	5′′	116.5	6.76 (br d, 7.8)
1′	127.7	-	6′′	125.4	7.11 (br d, 7.8)
2′	115.1	6.93 (br s)	7′′	129.9	7.35 (s)
3′	146.4	-	8′′	138.8	-
4′	151.4	-	9′′	165.9	-
5′	118.4	6.94 (d, 7.8)			
9-OCH_3_	52.9	3.67 (s)	9′′-OCH_3_	52.9	3.74 (s)

## 3. Experimental

### 3.1. General

Optical rotations were obtained on a Perkin-Elmer 341 digital polarimeter. UV and IR spectra were recorded on a Shimadzu UV2550 and FTIR-8400S spectrometer, respectively. One-dimensional (^1^H, ^13^C-APT) and two-dimensional (^1^H-^1^H COSY, HSQC, HMBC) NMR experiments were performed on a Bruker AV Ш 600 spectrometer operating at 600 MHz (^1^H) and 150 MHz (^13^C). HR-ESIMS spectra were performed on a LTQ-Obitrap XL spectrometer. The detection of all the compounds was achieved in ESI modes. C_18_ reversed-phase silica gel (40–63 μm, Merck, Darmstadt, Germany), Sephadex LH–20 (Pharmacia, Uppsala, Sweden) were used for the column chromatography. Precoated silica gel of GF_254_ plates (Zhi Fu Huang Wu Pilot Plant of Silica Gel Development, Yantai, China) were used for TLC. All solvents used were of analytical grade (Beijing Chemical Works, Beijing, China). Preparative HPLC was performed on a LUMTECH instrument with UV detector at 254 nm and using an YMC-Pack C_18_ column (250 mm × 20 mm inside diameter (I.D), 5 μm, YMC, Tokyo, Japan).

### 3.2. Plant Material

The *Clerodendranthus spicatu* sample was collected in Jinghong, Yunnan, China. A voucher specimen has been deposited in Institute of Medicinal Plant Development, Chinese Academy of Medical Sciences and Peking Union Medical College, Beijing, China.

### 3.3. Extraction and Isolation

The whole air-dried and powered plant material (10 Kg) was exhaustively extracted three times with water (80 L) at 70 °C for 30 min. The aqueous extract was evaporated to dryness under reduced pressure, followed by dissolution in hot water. After filtration, the filtrate was eluted on a macroporous resin column with water, 30%, 60% and 90% ethanol. The 90% ethanol eluate was further separated by Sephadex LH-20 column chromatography, eluted with methanol, to afford six fractions A–F. Fraction A was chromatographed by semi-preparative HPLC using MeOH–H_2_O (44:56) as mobile phase (flow rate 2 mL/min) to yield rosmarinic acid (**2**), methyl rosmarinate (**3**). Fraction C obtained by elution with methanol-water was further purified by reversed phase C_18_ gel column chromatography using MeOH-H_2_O with a gradient of 10:90 to 40:60 to give caffeic acid (**4**), methyl caffeate (**5**), ethyl caffeate (**6**). Fraction F was chromatographed on semi-preparative HPLC using MeOH–H_2_O (44:56) as mobile phase (flow rate 2 mL/min) to yield clerodendranoic acid (**1**).

### 3.4. Spectral Data

Compound **1**: brown powder. [α]_D_^25^+100.0° (*c* 1.0, MeOH); UV (MeOH) *λ*_max_ nm (log *ε*) 233 (0.41), 327 (0.57); IR (KBr) *ν*_max_ 3402, 2954, 1695, 1603, 1281, 1170, 1020, 817 cm^–1^; for ^1^H and ^13^C-NMR spectroscopic data, see Table 1; ESI-MS, *m/z* 589 [M+Na]^+^; HRESIMS, *m/z* 589.1321 [M+Na]^+^, calcd for C_29_H_26_O_12_, 589.1322. The structures of compounds **2**–**6** were identified by comparison of their spectral data with those reported in the literature. 

## 4. Conclusions

A new phenolic acid, 3′-*O*-(8′′-*Z*-methylcaffeoyl)-rosmarinic acid methyl ester, named clerodendranoic acid (**1**), together with five known ones, including rosmarinic acid (**2**), methyl rosmarinate (**3**), caffeic acid (**4**), methyl caffeate (**5**), ethyl caffeate (**6**), was isolated from the aerial parts of *C. spicatus*. 3′-*O*-(8′′-*Z*-methylcaffeoyl)-rosmarinic acid methyl ester is a new rosmarinic acid derivative and enriches the molecular diversity of this class. This finding represents an addition to the ongoing research on the pharmacological activity of the new compound, which may be helpful to understand the use of this plant in traditional medicine and should continue to clarify its actual health benefits.
